# Crystal structure of di-μ-tri­hydro­(penta­fluoro­phenyl)­borato-tetra­kis­(tetra­hydro­furan)­disodium

**DOI:** 10.1107/S2056989019017201

**Published:** 2020-01-07

**Authors:** Ryo Tanaka, Takeshi Shiono

**Affiliations:** a Department of Applied Chemistry, Graduate School of Engineering, Hiroshima University, 1-4-1 Kagamiyama, Higashi-hiroshima 739-8527, Japan

**Keywords:** organotri­hydro­borate, borohydride, sodium salt, crystal structure

## Abstract

The title compound, [Na(μ-C_6_F_5_BH_3_)(C_4_H_8_O)_2_]_2_, represents a dimeric structure of sodium and organoborohydride, located about a centre of inversion. The Na⋯B distances of 2.7845 (19) and 2.7494 (18) Å were apparently longer than the Li⋯B distances (2.403–2.537 Å) of the lithium organotri­hydro­borates in the previous reports. Moreover, an inter­action between the sodium atom and one fluorine atom on the 2-position of the benzene ring is observed [Na—F = 2.6373 (12) Å]. In the crystal, the dimeric mol­ecules are stacked along the *b*-axis *via* a π–π inter­action between the benzene rings.

## Abstract 2

The title compound, [Na_2_(μ-C_6_F_5_BH_3_)_2_(C_4_H_8_O)_4_], represents a dimeric structure of sodium and organoborohydride, located about a centre of inversion. In the crystal, the mol­ecules are stacked along the *b* axis *via* a π–π inter­action between the benzene rings.

## Chemical context   

A series of alkali-metal borohydride salts are known as the most important, reliable and commercially available reducing agents, especially for carbonyl compounds (Magano & Dunetz, 2012 ▸). The reducing ability of borohydrides can easily be tuned by introducing functional groups on boron or by changing their counter-cation. To understand the relationship between reactivity and composition of borohydride species, structural understandings based on crystallographic analysis would be important. The structures of these borohydride compounds are largely affected by the number of hydrides, bulkiness of substituents on boron, and metal. For example, sodium tri­ethyl­mono­hydro­borate forms a cubic tetra­mer (Bell *et al.*, 1980 ▸) and lithium tri­hydro­borate with a bulky alkyl group on boron gives a monomeric structure (Eaborn *et al.*, 1984 ▸). Reports of the structures of sodium alk­yl/aryl­tri­hydro­borates are very scarce, although some dimeric lithium organotri­hydro­borates (Knizek *et al.*, 2000 ▸; Franz *et al.*, 2011 ▸; Pospiech *et al.*, 2015 ▸; Murosaki *et al.*, 2016 ▸), monomeric lithium organotri­hydro­borate (Molitor & Gessner, 2013 ▸) and potassium aryl­tri­hydro­botate (Kaese *et al.*, 2016 ▸) have previously been characterized by X-ray crystal analyses. The only example of structurally characterized sodium alkyl­tri­hydro­borate is a compound bearing three meth­oxy­eth­oxy groups, and no inter­action between the hydrides and the sodium atom was observed in this case, because the sodium cation is trapped into the cage structure of the meth­oxy­eth­oxy groups and no longer forms contacts with the borohydride anion (Thalangamaarachchige *et al.*, 2019 ▸).

Herein, we report the first crystal structure analysis of sodium aryl­tri­hydro­borate, which bears a penta­fluoro­phenyl substituent on the boron centre.

## Structural commentary   

**Figure 1:**
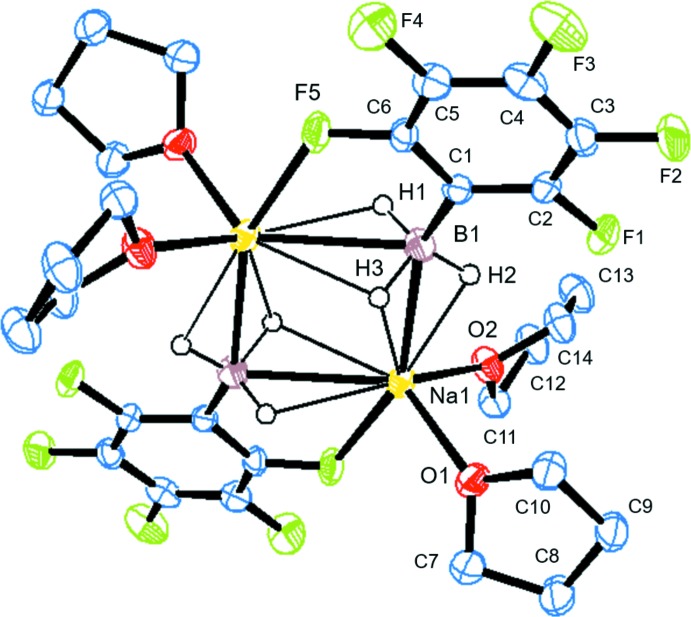
The mol­ecular structure of the title compound, with atom labels and 50% probability displacement ellipsoids for non-H atoms. H atoms other than hydrides have been omitted for clarity.

The title compound (Fig. 1 ▸) represents a dimeric structure bridged *via* three Na—H—B bonds, being located about a centre of inversion. The Na⋯B distances of 2.7845 (19) and 2.7494 (18) Å are apparently longer than the sum of covalent bond radii of sodium and boron (2.50 Å; Cordero *et al.*, 2008 ▸) and the previously reported lithium–boron distances (2.403–2.537 Å) in the lithium organotri­hydro­borates (Knizek *et al.*, 2000 ▸; Franz *et al.*, 2011 ▸; Pospiech *et al.*, 2015 ▸; Murosaki *et al.*, 2016 ▸). The Na⋯H distances show that one hydride (H3) binds to both sodium atoms of the dimer [Na1⋯H3 = 2.47 (2) Å and Na1^i^⋯H3 = 2.40 (2) Å; symmetry code: (i) −*x* + 1, −*y*, −*z*] while the other two hydrides (H1 and H2) bind only to one sodium atom [Na1^i^⋯1 = 2.34 (2) Å and Na1⋯H2 = 2.34 (3) Å]. Such a chelation mode was also observed in the previously reported dimeric structure of lithium tri­hydro­borates.
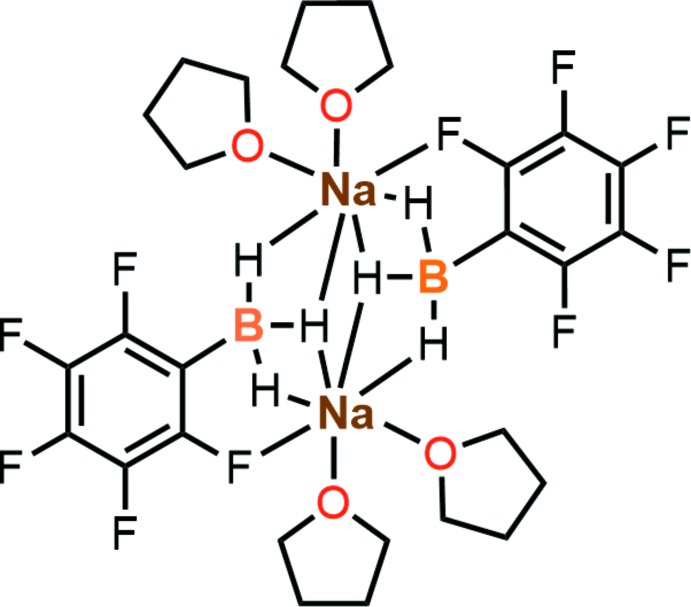


The distance between the sodium atom and fluorine atom F5 at the 2-position on the benzene ring [Na1^i^—F5 = 2.6373 (12) Å] is much shorter than the sum of van der Waals radii (3.74 Å), indicating the presence of a sodium–halogen inter­action. Such a halogen–metal inter­action is also observed in bromoaryl-substituted lithium tri­hydro­borate (Seven *et al.*, 2014 ▸). The environment around the sodium atom can therefore be seen as having a distorted trigonal–bypiramidal geometry with one fluorine atom, two boron atoms and two THF mol­ecules. The C—B bond [C1—B1 = 1.614 (2) Å] is significantly longer than the previously reported C—B bond lengths of lithium organotri­hydro­borates (1.597–1.613 Å), probably because of the electron-withdrawing property of the C_6_F_5_ group.

## Supra­molecular features   

**Figure 2:**
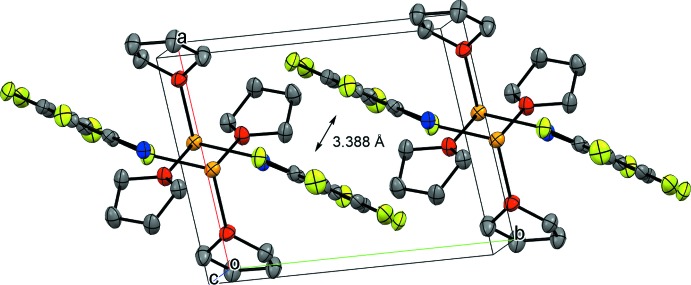
A packing diagram of the title compound, viewed approximately down the *c* axis, showing the π–π inter­action between the C_6_F_5_ rings. H atoms have been omitted.

In the crystal, the dimeric mol­ecules are stacked along the *b* axis *via* a π–π inter­action between the neighbouring C_6_F_5_ rings as shown in Fig. 2 ▸. The plane-to-plane distance, the centroid-to-centroid distance and the slippage are 3.388 (4), 3.582 (2) and 1.160 Å, respectively. The C_6_F_5_ rings are stacked in an anti-parallel manner, so that the boron atom on one C_6_F_5_ ring is close to the fluorine atom at 4-position on the other ring. However, the B⋯F distance [B1⋯F3^ii^ = 3.589 (2) Å; symmetry code: (ii) −*x* + 1, −*y* − 1, −*z*] is slightly longer than the sum of van der Waals radii (3.39 Å), suggesting that the B⋯F inter­action is weak. The distance between the closest hydrogen atom (H4) and centroid of the C_6_F_5_ ring is 3.343 Å, indicating the absence of C—H⋯π inter­actions.

## Database survey   

As described above, there is only one example of structural analysis on a sodium alkyl­tris­boronate complex (Thalangamaarachchige *et al.*, 2019 ▸). This complex exhibits a monomeric twitterionic structure without any inter­action between the borohydride and the sodium atom. Other examples of sodium tri­hydro­borates bearing a carbon-based substituent on boron, the sodium salt of boranocarbamates (Pitchumony *et al.*, 2010 ▸), cyano­borohydride (Custelcean *et al.*, 1998 ▸, 2002 ▸) and (iso­thio­cyanato)­tri­hydro­borate (Nöth & Warchhold, 2004 ▸) have been structurally characterized by X-ray crystallographic analyses. In these salts, the sodium cation exists as an adduct of polyethers or polyamine and is located distant from the borohydride anion.

## Synthesis and crystallization   

The title compound was prepared by the reaction of NaH (60% oil dispersion, 1.21 g, 30 mmol, washed twice with hexane prior to use) and (C_6_F_5_)BH_2_·S(CH_3_)_2_ (2.10 g, 8.7 mmol) in THF (20 mL) at 333 K for 5 h. The supernatant solution of the reaction mixture was separated and dried under vacuum. The obtained colourless solid was redissolved into 1 mL of THF, and 10 mL of hexane was layered on it. This solution was stored at 243 K overnight and 1.55 g (51%) of colourless crystals were obtained. ^19^F NMR (C_6_D_6_, 470 MHz): *δ* −134.72 (*br*, 2F), −162.85 (*t*, *J* = 20 Hz, 1F), −165.17 (*m*, 2F); ^11^B NMR (C_6_D_6_, 160 MHz): *δ* −36.71 (*q*, *J* = 86 Hz).

## Refinement   

Crystal data, data collection and structure refinement details are summarized in Table 1 ▸. All H atoms were located in a difference-Fourier map. The tetra­hydro­furan H atoms were refined using a riding model (C—H = 0.99 Å) with *U*_iso_(H) = 1.2*U*_eq_(C), while the H atoms on boron were refined isotrop­ically [refined B—H = 1.08 (3)–1.13 (2) Å].

## Supplementary Material

Crystal structure: contains datablock(s) global, I. DOI: 10.1107/S2056989019017201/is5529sup1.cif

Structure factors: contains datablock(s) I. DOI: 10.1107/S2056989019017201/is5529Isup3.hkl

CCDC reference: 1973896

Additional supporting information:  crystallographic information; 3D view; checkCIF report

## Figures and Tables

**Table 1:** Experimental details

Crystal data	
Chemical formula	[Na_2_(C_6_F_5_BH_3_)_2_(C_4_H_8_O)_4_]
*M*_r_	696.18
Crystal system, space group	Triclinic, *P* 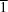
Temperature (K)	123
*a*, *b*, *c* (Å)	7.9698 (5), 10.1104 (6), 11.5208 (7)
α, β, γ (°)	113.461 (2), 105.685 (3), 91.805 (2)
*V* (Å^3^)	809.63 (9)
*Z*	1
Radiation type	Mo *K*α
μ (mm^−1^)	0.15
Crystal size (mm)	0.60 × 0.20 × 0.20

Data collection	
Diffractometer	Bruker APEXII CCD
Absorption correction	Multi-scan (*SADABS*; Bruker, 2016 ▸)
*T*_min_, *T*_max_	0.512, 0.746
No. of measured, independent and observed [*I* > 2σ(*I*)] reflections	4506, 3471, 3068
*R*_int_	0.032
(sin θ/λ)_max_ (Å^−1^)	0.648

Refinement	
*R*[*F* ^2^ > 2σ(*F* ^2^)], *wR*(*F* ^2^), *S*	0.047, 0.146, 1.08
No. of reflections	3471
No. of parameters	220
H-atom treatment	H atoms treated by a mixture of independent and constrained refinement
Δρ_max_, Δρ_min_ (e Å^−3^)	0.31, −0.45

Computer programs: *APEX2* and *SAINT* (Bruker, 2012 ▸), *SIR97* (Altomare *et al.*, 1999 ▸), *SHELXL2014* (Sheldrick, 2015 ▸), *Mercury* (Macrae *et al.*, 2006 ▸) and *SHELXTL* (Sheldrick, 2015 ▸).

## supplementary crystallographic information

### Crystal data

**Table:**

[Na_2_(C_6_F_5_BH_3_)_2_(C_4_H_8_O)_4_]	*Z* = 1
*M**_r_* = 696.18	*F*(000) = 360
Triclinic, *P*1	*D*_x_ = 1.428 Mg m^−^^3^
Hall symbol: -P 1	Mo *K*α radiation, λ = 0.71069 Å
*a* = 7.9698 (5) Å	Cell parameters from 4126 reflections
*b* = 10.1104 (6) Å	θ = 2.7–27.4°
*c* = 11.5208 (7) Å	µ = 0.15 mm^−^^1^
α = 113.461 (2)°	*T* = 123 K
β = 105.685 (3)°	Block, colourless
γ = 91.805 (2)°	0.60 × 0.20 × 0.20 mm
*V* = 809.63 (9) Å^3^	

### Data collection

**Table:**

Bruker APEXII CCD diffractometer	3068 reflections with *I* > 2σ(*I*)
φ and ω scans	*R*_int_ = 0.032
Absorption correction: multi-scan (SADABS; Bruker, 2016)	θ_max_ = 27.4°, θ_min_ = 2.0°
*T*_min_ = 0.512, *T*_max_ = 0.746	*h* = −10→8
4506 measured reflections	*k* = −13→12
3471 independent reflections	*l* = −14→12

### Refinement

**Table:**

Refinement on *F*^2^	0 restraints
Least-squares matrix: full	Primary atom site location: structure-invariant direct methods
*R*[*F*^2^ > 2σ(*F*^2^)] = 0.047	H atoms treated by a mixture of independent and constrained refinement
*wR*(*F*^2^) = 0.146	*w* = 1/[σ^2^(*F*_o_^2^) + (0.0819*P*)^2^ + 0.2169*P*] where *P* = (*F*_o_^2^ + 2*F*_c_^2^)/3
*S* = 1.08	(Δ/σ)_max_ < 0.001
3471 reflections	Δρ_max_ = 0.31 e Å^−^^3^
220 parameters	Δρ_min_ = −0.45 e Å^−^^3^

### Special details

**Table:**

Geometry. All esds (except the esd in the dihedral angle between two l.s. planes) are estimated using the full covariance matrix. The cell esds are taken into account individually in the estimation of esds in distances, angles and torsion angles; correlations between esds in cell parameters are only used when they are defined by crystal symmetry. An approximate (isotropic) treatment of cell esds is used for estimating esds involving l.s. planes.
Refinement. Reflections were merged by SHELXL according to the crystal class for the calculation of statistics and refinement. _reflns_Friedel_fraction is defined as the number of unique Friedel pairs measured divided by the number that would be possible theoretically, ignoring centric projections and systematic absences.

### Fractional atomic coordinates and isotropic or equivalent isotropic displacement parameters (Å^2^)

**Table:**

	*x*	*y*	*z*	*U*_iso_*/*U*_eq_	
NA1	0.55346 (7)	−0.03356 (7)	−0.15797 (5)	0.02597 (18)	
B1	0.5516 (2)	−0.20185 (19)	−0.02053 (16)	0.0272 (3)	
H1	0.412 (3)	−0.193 (2)	−0.022 (2)	0.043 (6)*	
H2	0.560 (3)	−0.244 (3)	−0.120 (2)	0.053 (6)*	
H3	0.638 (3)	−0.092 (2)	0.037 (2)	0.037 (5)*	
C1	0.63209 (18)	−0.30571 (15)	0.04965 (13)	0.0217 (3)	
C2	0.71559 (19)	−0.42272 (17)	−0.00498 (14)	0.0256 (3)	
C3	0.78844 (19)	−0.50723 (17)	0.05860 (16)	0.0296 (3)	
C4	0.7778 (2)	−0.47796 (18)	0.18383 (16)	0.0310 (3)	
C5	0.6944 (2)	−0.36360 (18)	0.24263 (14)	0.0296 (3)	
C6	0.62500 (19)	−0.28259 (16)	0.17530 (13)	0.0238 (3)	
F1	0.72989 (14)	−0.45982 (12)	−0.12796 (10)	0.0395 (3)	
F2	0.86728 (14)	−0.61935 (12)	−0.00036 (13)	0.0453 (3)	
F3	0.84800 (15)	−0.55697 (12)	0.24888 (12)	0.0469 (3)	
F4	0.68262 (17)	−0.33360 (13)	0.36492 (10)	0.0475 (3)	
F5	0.54502 (14)	−0.17008 (11)	0.23880 (9)	0.0343 (2)	
O1	0.83333 (15)	−0.03501 (13)	−0.16984 (12)	0.0327 (3)	
C7	0.9190 (3)	0.0619 (2)	−0.2058 (2)	0.0436 (4)	
H4	1.016334	0.130984	−0.128474	0.052*	
H5	0.834348	0.118300	−0.238397	0.052*	
C8	0.9905 (3)	−0.0347 (2)	−0.31551 (19)	0.0430 (4)	
H6	0.911623	−0.050243	−0.404062	0.052*	
H7	1.109827	0.009804	−0.303714	0.052*	
C9	0.9967 (3)	−0.1771 (2)	−0.30160 (18)	0.0391 (4)	
H8	1.118106	−0.200710	−0.286654	0.047*	
H9	0.917077	−0.258235	−0.382236	0.047*	
C10	0.9356 (2)	−0.1511 (2)	−0.18186 (17)	0.0348 (4)	
H10	0.862781	−0.240318	−0.195466	0.042*	
H11	1.037924	−0.122799	−0.100803	0.042*	
O2	0.40793 (15)	−0.18090 (12)	−0.37962 (10)	0.0319 (3)	
C11	0.3406 (2)	−0.15236 (19)	−0.49440 (15)	0.0328 (4)	
H12	0.434473	−0.146717	−0.534402	0.039*	
H13	0.292566	−0.059350	−0.470737	0.039*	
C12	0.1955 (3)	−0.2805 (2)	−0.59003 (16)	0.0392 (4)	
H14	0.192110	−0.307211	−0.683241	0.047*	
H15	0.078771	−0.257016	−0.580757	0.047*	
C13	0.2449 (3)	−0.4036 (2)	−0.54953 (17)	0.0411 (4)	
H16	0.149650	−0.439183	−0.524004	0.049*	
H17	0.267784	−0.486333	−0.623172	0.049*	
C14	0.4109 (2)	−0.33571 (19)	−0.43176 (16)	0.0362 (4)	
H18	0.410810	−0.372273	−0.363810	0.043*	
H19	0.517132	−0.358754	−0.460167	0.043*	

### Atomic displacement parameters (Å^2^)

**Table:**

	*U*^11^	*U*^22^	*U*^33^	*U*^12^	*U*^13^	*U*^23^
NA1	0.0285 (3)	0.0299 (3)	0.0210 (3)	0.0070 (2)	0.0097 (2)	0.0107 (2)
B1	0.0357 (8)	0.0252 (8)	0.0217 (7)	0.0061 (7)	0.0080 (6)	0.0114 (6)
C1	0.0235 (6)	0.0215 (7)	0.0192 (6)	0.0010 (5)	0.0062 (5)	0.0080 (5)
C2	0.0297 (7)	0.0240 (7)	0.0229 (7)	0.0024 (6)	0.0130 (5)	0.0066 (6)
C3	0.0259 (7)	0.0224 (8)	0.0382 (8)	0.0058 (6)	0.0113 (6)	0.0093 (6)
C4	0.0293 (7)	0.0274 (8)	0.0332 (8)	0.0004 (6)	−0.0014 (6)	0.0171 (7)
C5	0.0366 (8)	0.0312 (8)	0.0177 (6)	−0.0010 (6)	0.0037 (6)	0.0106 (6)
C6	0.0284 (7)	0.0218 (7)	0.0184 (6)	0.0041 (5)	0.0084 (5)	0.0050 (5)
F1	0.0575 (6)	0.0367 (6)	0.0321 (5)	0.0129 (5)	0.0304 (5)	0.0110 (4)
F2	0.0441 (6)	0.0295 (6)	0.0667 (7)	0.0184 (4)	0.0277 (5)	0.0168 (5)
F3	0.0480 (6)	0.0391 (6)	0.0509 (6)	0.0056 (5)	−0.0054 (5)	0.0298 (5)
F4	0.0726 (8)	0.0513 (7)	0.0204 (5)	0.0076 (6)	0.0123 (5)	0.0184 (5)
F5	0.0497 (6)	0.0299 (5)	0.0264 (4)	0.0135 (4)	0.0208 (4)	0.0085 (4)
O1	0.0319 (6)	0.0343 (6)	0.0402 (6)	0.0103 (5)	0.0189 (5)	0.0187 (5)
C7	0.0532 (11)	0.0327 (9)	0.0599 (12)	0.0128 (8)	0.0352 (9)	0.0226 (9)
C8	0.0622 (11)	0.0384 (10)	0.0445 (10)	0.0171 (9)	0.0315 (9)	0.0232 (8)
C9	0.0544 (10)	0.0337 (9)	0.0390 (9)	0.0150 (8)	0.0266 (8)	0.0166 (7)
C10	0.0419 (9)	0.0367 (9)	0.0344 (8)	0.0142 (7)	0.0170 (7)	0.0195 (7)
O2	0.0415 (6)	0.0265 (6)	0.0211 (5)	0.0046 (5)	0.0035 (4)	0.0078 (4)
C11	0.0446 (9)	0.0298 (8)	0.0251 (7)	0.0061 (7)	0.0098 (6)	0.0134 (6)
C12	0.0491 (10)	0.0314 (9)	0.0259 (7)	0.0065 (7)	0.0001 (7)	0.0083 (7)
C13	0.0553 (11)	0.0288 (9)	0.0288 (8)	0.0015 (8)	0.0046 (7)	0.0076 (7)
C14	0.0506 (10)	0.0272 (8)	0.0280 (7)	0.0116 (7)	0.0077 (7)	0.0113 (6)

### Geometric parameters (Å, º)

**Table:**

NA1—O1	2.2714 (12)	O1—C10	1.433 (2)
NA1—O2	2.3122 (12)	C7—C8	1.522 (3)
NA1—F5^i^	2.6373 (12)	C7—H4	0.9900
NA1—B1	2.7494 (18)	C7—H5	0.9900
NA1—B1^i^	2.7845 (19)	C8—C9	1.511 (3)
NA1—NA1^i^	3.7658 (11)	C8—H6	0.9900
NA1—H2	2.33 (2)	C8—H7	0.9900
NA1—H3	2.47 (2)	C9—C10	1.514 (2)
B1—C1	1.614 (2)	C9—H8	0.9900
B1—NA1^i^	2.7844 (19)	C9—H9	0.9900
B1—H1	1.11 (2)	C10—H10	0.9900
B1—H2	1.08 (3)	C10—H11	0.9900
B1—H3	1.13 (2)	O2—C11	1.4317 (18)
C1—C2	1.386 (2)	O2—C14	1.440 (2)
C1—C6	1.3889 (18)	C11—C12	1.520 (2)
C2—F1	1.3520 (16)	C11—H12	0.9900
C2—C3	1.380 (2)	C11—H13	0.9900
C3—F2	1.3431 (18)	C12—C13	1.522 (3)
C3—C4	1.380 (2)	C12—H14	0.9900
C4—F3	1.3384 (18)	C12—H15	0.9900
C4—C5	1.380 (2)	C13—C14	1.514 (2)
C5—F4	1.3486 (17)	C13—H16	0.9900
C5—C6	1.371 (2)	C13—H17	0.9900
C6—F5	1.3677 (17)	C14—H18	0.9900
F5—NA1^i^	2.6373 (12)	C14—H19	0.9900
O1—C7	1.425 (2)		
			
O1—NA1—O2	97.78 (5)	F5—C6—C5	116.66 (13)
O1—NA1—F5^i^	101.16 (4)	F5—C6—C1	118.44 (13)
O2—NA1—F5^i^	80.88 (4)	C5—C6—C1	124.90 (14)
O1—NA1—B1	100.56 (5)	C6—F5—NA1^i^	124.58 (8)
O2—NA1—B1	107.00 (5)	C7—O1—C10	105.86 (12)
F5^i^—NA1—B1	155.60 (5)	C7—O1—NA1	124.28 (11)
O1—NA1—B1^i^	123.38 (5)	C10—O1—NA1	127.60 (10)
O2—NA1—B1^i^	129.23 (5)	O1—C7—C8	105.75 (15)
F5^i^—NA1—B1^i^	64.36 (4)	O1—C7—H4	110.6
B1—NA1—B1^i^	94.23 (5)	C8—C7—H4	110.6
O1—NA1—NA1^i^	122.73 (4)	O1—C7—H5	110.6
O2—NA1—NA1^i^	132.93 (4)	C8—C7—H5	110.6
F5^i^—NA1—NA1^i^	110.18 (3)	H4—C7—H5	108.7
B1—NA1—NA1^i^	47.51 (4)	C9—C8—C7	104.83 (14)
B1^i^—NA1—NA1^i^	46.73 (4)	C9—C8—H6	110.8
O1—NA1—H2	91.5 (6)	C7—C8—H6	110.8
O2—NA1—H2	87.9 (6)	C9—C8—H7	110.8
F5^i^—NA1—H2	164.1 (6)	C7—C8—H7	110.8
B1—NA1—H2	22.6 (6)	H6—C8—H7	108.9
B1^i^—NA1—H2	116.2 (6)	C8—C9—C10	104.49 (14)
NA1^i^—NA1—H2	69.7 (6)	C8—C9—H8	110.9
O1—NA1—H3	91.0 (5)	C10—C9—H8	110.9
O2—NA1—H3	130.7 (5)	C8—C9—H9	110.9
F5^i^—NA1—H3	144.5 (5)	C10—C9—H9	110.9
B1—NA1—H3	24.2 (5)	H8—C9—H9	108.9
B1^i^—NA1—H3	81.0 (5)	O1—C10—C9	105.64 (13)
NA1^i^—NA1—H3	38.6 (5)	O1—C10—H10	110.6
H2—NA1—H3	43.3 (8)	C9—C10—H10	110.6
C1—B1—NA1	155.20 (11)	O1—C10—H11	110.6
C1—B1—NA1^i^	109.90 (9)	C9—C10—H11	110.6
NA1—B1—NA1^i^	85.77 (5)	H10—C10—H11	108.7
C1—B1—H1	111.0 (11)	C11—O2—C14	104.91 (11)
NA1—B1—H1	93.7 (11)	C11—O2—NA1	133.50 (10)
NA1^i^—B1—H1	55.7 (12)	C14—O2—NA1	119.96 (9)
C1—B1—H2	109.9 (13)	O2—C11—C12	105.00 (13)
NA1—B1—H2	56.3 (13)	O2—C11—H12	110.7
NA1^i^—B1—H2	140.1 (13)	C12—C11—H12	110.7
H1—B1—H2	110.2 (17)	O2—C11—H13	110.7
C1—B1—H3	107.0 (11)	C12—C11—H13	110.7
NA1—B1—H3	64.1 (11)	H12—C11—H13	108.8
NA1^i^—B1—H3	58.4 (11)	C11—C12—C13	104.30 (14)
H1—B1—H3	111.4 (16)	C11—C12—H14	110.9
H2—B1—H3	107.2 (16)	C13—C12—H14	110.9
C2—C1—C6	113.31 (13)	C11—C12—H15	110.9
C2—C1—B1	125.13 (12)	C13—C12—H15	110.9
C6—C1—B1	121.55 (13)	H14—C12—H15	108.9
F1—C2—C3	115.87 (14)	C14—C13—C12	104.48 (14)
F1—C2—C1	119.98 (13)	C14—C13—H16	110.9
C3—C2—C1	124.15 (14)	C12—C13—H16	110.9
F2—C3—C2	121.03 (15)	C14—C13—H17	110.9
F2—C3—C4	119.32 (15)	C12—C13—H17	110.9
C2—C3—C4	119.64 (15)	H16—C13—H17	108.9
F3—C4—C5	119.96 (15)	O2—C14—C13	105.39 (14)
F3—C4—C3	121.29 (16)	O2—C14—H18	110.7
C5—C4—C3	118.75 (15)	C13—C14—H18	110.7
F4—C5—C6	121.25 (15)	O2—C14—H19	110.7
F4—C5—C4	119.52 (15)	C13—C14—H19	110.7
C6—C5—C4	119.23 (14)	H18—C14—H19	108.8
			
NA1—B1—C1—C2	−45.4 (3)	F4—C5—C6—C1	179.69 (13)
NA1^i^—B1—C1—C2	−172.01 (11)	C4—C5—C6—C1	−0.3 (2)
NA1—B1—C1—C6	133.6 (2)	C2—C1—C6—F5	−179.83 (12)
NA1^i^—B1—C1—C6	7.05 (16)	B1—C1—C6—F5	1.0 (2)
C6—C1—C2—F1	178.94 (12)	C2—C1—C6—C5	0.9 (2)
B1—C1—C2—F1	−1.9 (2)	B1—C1—C6—C5	−178.22 (14)
C6—C1—C2—C3	−1.3 (2)	C5—C6—F5—NA1^i^	169.12 (10)
B1—C1—C2—C3	177.85 (14)	C1—C6—F5—NA1^i^	−10.18 (17)
F1—C2—C3—F2	−0.3 (2)	C10—O1—C7—C8	35.26 (19)
C1—C2—C3—F2	179.89 (13)	NA1—O1—C7—C8	−128.73 (14)
F1—C2—C3—C4	−179.20 (13)	O1—C7—C8—C9	−19.7 (2)
C1—C2—C3—C4	1.0 (2)	C7—C8—C9—C10	−2.2 (2)
F2—C3—C4—F3	1.6 (2)	C7—O1—C10—C9	−36.82 (18)
C2—C3—C4—F3	−179.52 (13)	NA1—O1—C10—C9	126.48 (13)
F2—C3—C4—C5	−179.17 (13)	C8—C9—C10—O1	23.29 (19)
C2—C3—C4—C5	−0.3 (2)	C14—O2—C11—C12	39.54 (17)
F3—C4—C5—F4	−0.8 (2)	NA1—O2—C11—C12	−155.60 (12)
C3—C4—C5—F4	179.93 (13)	O2—C11—C12—C13	−24.77 (18)
F3—C4—C5—C6	179.21 (13)	C11—C12—C13—C14	1.6 (2)
C3—C4—C5—C6	−0.1 (2)	C11—O2—C14—C13	−38.57 (18)
F4—C5—C6—F5	0.4 (2)	NA1—O2—C14—C13	154.06 (11)
C4—C5—C6—F5	−179.57 (13)	C12—C13—C14—O2	21.94 (19)

Symmetry code: (i) −*x*+1, −*y*, −*z*.
